# Supplementary material for: Prevalence of depression and its associated factors among adolescents in China during the early stage of the COVID-19 outbreak
Source: PeerJ. 2021 Nov 11;9:e12223. doi: 10.7717/peerj.12223 (PMC8590796; doi:10.7717/peerj.12223)
Supplement: Supplemental Information 2 [file peerj-09-12223-s002.docx]

Supplementary Table 1. Values of categories variables.

| **Variables** | **Values** |
| --- | --- |
| **Characteristics** |  |
| Gender | 1=male, 2=female |
| Residence | 1=home, 2=school dorm, 3=others |
| Grade | 1=grade one junior secondary school  2=grade two junior secondary school  3=grade three junior secondary school  4=grade one senior secondary school  5=grade two senior secondary school  6=grade three senior secondary school |
| Father as frontline worker | 1=yes, 2=no |
| Mother as frontline worker | 1=yes, 2=no |
| Having infected acquaintances | 1=yes, 2=no |
| Attitudes about COVID-19 | 1=very concern  2=general concern  3=not concern |
| Sleep duration/day | 1=<6h, 2=6-8h, 3=>8h |
| Frequency of outdoor activities/week | 1=never, 2=<1 time/week, 3=1-3 times/week, 4=>3 times/week |
| Study duration/day | 1=<4h, 2=4-8h, 3=8-12h, 4=>12h |
| Participant in distance learning | 1=yes, 2=no |
| Exercise duration/day | 1=<30min, 2=30-60min, 3=>60min |
| Living area (No. of infected patients) | 1=10-99  2=100-999  3=1000-9999  4=>10000 |
| **CES-D** |  |
| I was bothered by things that usually don't bother me | 1=Rarely or none of the time (less than 1 day)  2=Some or a little of the time (1-2 days)  3=Occasionally or a moderate amount of time (3-4 days)  4=Most or all of the time (5-7 days) |
| I did not feel like eating; my appetite was poor | 1=Rarely or none of the time (less than 1 day)  2=Some or a little of the time (1-2 days)  3=Occasionally or a moderate amount of time (3-4 days)  4=Most or all of the time (5-7 days) |
| I felt that I could not shake off the blues even with help from my family or friends | 1=Rarely or none of the time (less than 1 day)  2=Some or a little of the time (1-2 days)  3=Occasionally or a moderate amount of time (3-4 days)  4=Most or all of the time (5-7 days) |
| I felt I was as good as other kids | 1=Rarely or none of the time (less than 1 day)  2=Some or a little of the time (1-2 days)  3=Occasionally or a moderate amount of time (3-4 days)  4=Most or all of the time (5-7 days) |
| I had trouble keeping my mind on what I was doing | 1=Rarely or none of the time (less than 1 day)  2=Some or a little of the time (1-2 days)  3=Occasionally or a moderate amount of time (3-4 days)  4=Most or all of the time (5-7 days) |
| I felt depressed | 1=Rarely or none of the time (less than 1 day)  2=Some or a little of the time (1-2 days)  3=Occasionally or a moderate amount of time (3-4 days)  4=Most or all of the time (5-7 days) |
| I felt that everything I did was an effort | 1=Rarely or none of the time (less than 1 day)  2=Some or a little of the time (1-2 days)  3=Occasionally or a moderate amount of time (3-4 days)  4=Most or all of the time (5-7 days) |
| I felt hopeful about the future | 1=Rarely or none of the time (less than 1 day)  2=Some or a little of the time (1-2 days)  3=Occasionally or a moderate amount of time (3-4 days)  4=Most or all of the time (5-7 days) |
| I thought my life had been a failure | 1=Rarely or none of the time (less than 1 day)  2=Some or a little of the time (1-2 days)  3=Occasionally or a moderate amount of time (3-4 days)  4=Most or all of the time (5-7 days) |
| I felt fearful | 1=Rarely or none of the time (less than 1 day)  2=Some or a little of the time (1-2 days)  3=Occasionally or a moderate amount of time (3-4 days)  4=Most or all of the time (5-7 days) |
| My sleep was restless | 1=Rarely or none of the time (less than 1 day)  2=Some or a little of the time (1-2 days)  3=Occasionally or a moderate amount of time (3-4 days)  4=Most or all of the time (5-7 days) |
| I was happy | 1=Rarely or none of the time (less than 1 day)  2=Some or a little of the time (1-2 days)  3=Occasionally or a moderate amount of time (3-4 days)  4=Most or all of the time (5-7 days) |
| I talked less than usual | 1=Rarely or none of the time (less than 1 day)  2=Some or a little of the time (1-2 days)  3=Occasionally or a moderate amount of time (3-4 days)  4=Most or all of the time (5-7 days) |
| I felt lonely | 1=Rarely or none of the time (less than 1 day)  2=Some or a little of the time (1-2 days)  3=Occasionally or a moderate amount of time (3-4 days)  4=Most or all of the time (5-7 days) |
| People were unfriendly | 1=Rarely or none of the time (less than 1 day)  2=Some or a little of the time (1-2 days)  3=Occasionally or a moderate amount of time (3-4 days)  4=Most or all of the time (5-7 days) |
| I enjoyed life | 1=Rarely or none of the time (less than 1 day)  2=Some or a little of the time (1-2 days)  3=Occasionally or a moderate amount of time (3-4 days)  4=Most or all of the time (5-7 days) |
| I had crying spells | 1=Rarely or none of the time (less than 1 day)  2=Some or a little of the time (1-2 days)  3=Occasionally or a moderate amount of time (3-4 days)  4=Most or all of the time (5-7 days) |
| I felt sad | 1=Rarely or none of the time (less than 1 day)  2=Some or a little of the time (1-2 days)  3=Occasionally or a moderate amount of time (3-4 days)  4=Most or all of the time (5-7 days) |
| I felt that people disliked me | 1=Rarely or none of the time (less than 1 day)  2=Some or a little of the time (1-2 days)  3=Occasionally or a moderate amount of time (3-4 days)  4=Most or all of the time (5-7 days) |
| I could not get going | 1=Rarely or none of the time (less than 1 day)  2=Some or a little of the time (1-2 days)  3=Occasionally or a moderate amount of time (3-4 days)  4=Most or all of the time (5-7 days) |
