# Supplementary material for: Prevalence of depression and its associated factors among adolescents in China during the early stage of the COVID-19 outbreak
Source: PeerJ. 2021 Nov 11;9:e12223. doi: 10.7717/peerj.12223 (PMC8590796; doi:10.7717/peerj.12223)
Supplement: Supplemental Information 3 [file peerj-09-12223-s003.pdf]

Questionnaire of Depression in Middle School Students during COVID-19 outbreak

1. Baseline Characteristics

(1). Gender：1=male　2=female

(2). Age：________years

(3). Birthplace：________Province/Municipality/Autonomous region

(4). Residence during the pandemic outbreak：1=home　2=school dorms 3=others

(5). Grades：Junior secondary school：1=grade 1　2=grade 2　3=grade 3

Senior secondary school：4=grade 1　5=grade 2　6=grade 3

2. Pandemic related factors

(1). Is your father a frontline worker?　1=yes　2=no

(2). is your mother a frontline worker?　1=yes　2=no

(3). Did you have any acquaintances been infected? 1=yes　2=no

(4). How concerned you are about the COVID-19 outbreak？

1=very concerned　2=general concerned　3=not concerned

3. Study related factors

(1). How many hours did you sleep every day in the last week?

1=<6h　2=6-8h　3=>8h

(2). How many hours did you study every day in the last week？

1=<4h　2=4-8h　3=8-12h　4>12h

(3). How about the frequency of outdoor activities in the last month?

1=never　2=<1 time/week　3=1-3 times/week　4=>4 times/week

(4). Did you participate in the distance learning?　1=yes　2=no

(5). How long did you exercise every day in the last week?　1=<30 min　2=30-60 min　3=>60 min

4. Depression symptoms investigation (CES-D)

Below is a list of some ways you may have felt or behaved. Please indicate how often you have felt this way during the last week by checking the appropriate space. Please only provide one answer to each question.

	**During the past week**:	***Rarely*** or none of the time (less than 1 day)	***Some*** or a *little* of the time (1-2 days)	***Occasionally*** or a moderate amount of time (3-4 days)	***Most*** or all of the time (5-7 days)
1.	I was bothered by things that usually don't bother me.				
2.	I did not feel like eating; my appetite was poor.				
3.	I felt that I could not shake off the blues even with help from my family or friends.				
4.	I felt I was just as good as other people.				
5.	I had trouble keeping my mind on what I was doing.				
6.	I felt depressed.				
7.	I felt that everything I did was an effort.				
8.	I felt hopeful about the future.				
9.	I thought my life had been a failure.				
10.	I felt fearful.				
11.	My sleep was restless.				
12.	I was happy.				
13.	I talked less than usual.				
14.	I felt lonely.				
15.	People were unfriendly.				
16.	I enjoyed life.				
17.	I had crying spells.				
18.	I felt sad.				
19.	I felt that people disliked me.				
20.	I could not get going.				

中学生新冠病毒疫情期间焦虑抑郁情况调查问卷

一、一般情况调查

1.您的性别：1=男　2=女

2.您的年龄：________岁

3.您的籍贯：________省/直辖市/自治区/特别行政区

4.疫情发生期间您主要居住地点：1=原籍家中　2=学校宿舍　3=其他

5.您的年级：初中：1=一年级　2=二年级　3=三年级

　　　　　　高中：4=一年级　5=二年级　6=三年级

二、疫情相关调查

1.您父亲是否参与到此次疫情的一线工作当中　1=是　2=否

2.您母亲是否参与到此次疫情的一线工作当中　1=是　2=否

3.您是否有家人、朋友等熟悉的人感染新型冠状病毒肺炎？1=是　2=否

4.您对此次疫情关注的程度？　1=非常关注　2=一般关注　3=不太关注

三、学习与生活习惯调查

1.您最近一周每天的睡眠时间是多少小时（白天+晚上）？

1=<6 小时　2=6-8 小时　3=>8 小时

2.您最近一周每天的学习时间是多少小时？

1=<4 小时　2=4-8 小时　3=8-12 小时　4>12 小时

3.您是否参加网络课程的学习？1=是　2=否

4.您最近一周每天参加体育锻炼的时间是多少？1=<30 分钟　2=30-60 分钟　3=>60 分钟

5.自疫情开始，您出门的频率是多少？

1=从来没有　2=<1 次/周　3=1-3 次/周　4=>3 次/周

四、抑郁调查

CES-DC

下面是你可能感觉或行为相关的情况列表，请检查最近一周您出现这种感受有多少。

最近一周

1. 平时并不困扰我的事情让我烦恼　0=根本没有　1=一点　2=有时　3=许多

2. 我不想吃东西，胃口不好　0=根本没有　1=一点　2=有时　3=许多

3. 我高兴不起来，即便是家人或朋友的帮助也无法好转

0=根本没有　1=一点　2=有时　3=许多

4. 我感觉自己和其他孩子一样好　0=根本没有　1=一点　2=有时　3=许多

5. 对我所做的事情，很难集中注意力　0=根本没有　1=一点　2=有时　3=许多

6. 我感到失落和不幸　0=根本没有　1=一点　2=有时　3=许多

7. 我觉得非常疲惫以至于不能做事　0=根本没有　1=一点　2=有时　3=许多

8. 我感到未来有希望　0=根本没有　1=一点　2=有时　3=许多

9. 我觉得以前做的事情都不对　0=根本没有　1=一点　2=有时　3=许多

10. 我感到恐惧　0=根本没有　1=一点　2=有时　3=许多

11. 我睡眠不如平时好　0=根本没有　1=一点　2=有时　3=许多

12. 我很幸福　0=根本没有　1=一点　2=有时　3=许多

13. 我比平时话少了　0=根本没有　1=一点　2=有时　3=许多

14. 我感到孤独，好像一个朋友都没有　0=根本没有　1=一点　2=有时　3=许多

15. 我认识的人对我不友好，好像不愿和我在一起

0=根本没有　1=一点　2=有时　3=许多

16. 我生活快乐　0=根本没有　1=一点　2=有时　3=许多

17. 我想哭　0=根本没有　1=一点　2=有时　3=许多

18. 我感到悲伤　0=根本没有　1=一点　2=有时　3=许多

19. 我觉得别人不喜欢我　0=根本没有　1=一点　2=有时　3=许多

20. 我启动做事困难　0=根本没有　1=一点　2=有时　3=许多
